# Prognostic value of preoperative high-sensitivity modified Glasgow prognostic score in advanced colon cancer: a retrospective observational study

**DOI:** 10.1186/s12885-021-09113-8

**Published:** 2022-01-03

**Authors:** Kenta Kasahara, Masanobu Enomoto, Ryutaro Udo, Tomoya Tago, Junichi Mazaki, Tetsuo Ishizaki, Tesshi Yamada, Yuichi Nagakawa, Kenji Katsumata, Akihiko Tsuchida

**Affiliations:** Department of Gastrointestinal and Pediatric Surgery, Tokyo Medical University, 6-7-1 Nishi Shinjuku, Shinjuku-ku, Tokyo, 160-0023 Japan

**Keywords:** Glasgow prognostic score, Modified Glasgow prognostic score, High-sensitivity modified Glasgow prognostic score, Colon cancer

## Abstract

**Background:**

Several studies have demonstrated that the preoperative Glasgow prognostic score (GPS) and modified GPS (mGPS) reflected the prognosis in patients undergoing curative surgery for colorectal cancer. However, there are no reports on long-term prognosis prediction using high-sensitivity mGPS (HS-GPS) in colorectal cancer. Therefore, this study aimed to calculate the prognostic value of preoperative HS-GPS in patients with colon cancer.

**Methods:**

A cohort of 595 patients with advanced resectable colon cancer managed at our institution was analysed retrospectively. HS-GPS, GPS, and mGPS were evaluated for their ability to predict prognosis based on overall survival (OS) and recurrence-free survival (RFS).

**Results:**

In the univariate analysis, HS-GPS was able to predict the prognosis with significant differences in OS but was not superior in assessing RFS. In the multivariate analysis of the HS-GPS model, age, pT, pN, and HS-GPS of 2 compared to HS-GPS of 0 (2 vs 0; hazard ratio [HR], 2.638; 95% confidence interval [CI], 1.046–6.650; *P* = 0.04) were identified as independent prognostic predictors of OS. In the multivariate analysis of the GPS model, GPS 2 vs 0 (HR, 1.444; 95% CI, 1.018–2.048; *P* = 0.04) and GPS 2 vs 1 (HR, 2.933; 95% CI, 1.209–7.144; *P* = 0.017), and in that of the mGPS model, mGPS 2 vs 0 (HR, 1.51; 95% CI, 1.066–2.140; *P* = 0.02) were independent prognostic predictors of OS. In each classification, GPS outperformed HS-GPS in predicting OS with a significant difference in the area under the receiver operating characteristic curve. In the multivariate analysis of the GPS model, GPS 2 vs 0 (HR, 1.537; 95% CI, 1.190–1.987; *P* = 0.002), and in that of the mGPS model, pN, CEA were independent prognostic predictors of RFS.

**Conclusion:**

HS-GPS is useful for predicting the prognosis of resectable advanced colon cancer. However, GPS may be more useful than HS-GPS as a prognostic model for advanced colon cancer.

## Background

Colorectal cancer (CRC) is the third most commonly diagnosed cancer and the second leading cause of cancer-related mortality worldwide [1]. CRC prognosis is based on the Union for International Cancer Control (UICC) tumour node metastasis (TNM) classification; however, differences in outcomes have been reported among patients presenting with the same disease stage [2, 3]. Concurrently, various inflammatory biomarkers have been suggested as relevant survival predictors in this patient group [4, 5].

Several studies have demonstrated that preoperative Glasgow prognostic score (GPS) and modified GPS (mGPS) reflected the prognosis in patients with CRC or colon cancer (CC) who were undergoing curative surgery [2–4]. It was reported that high-sensitivity mGPS (HS-GPS) was useful for changing the cut-off value of GPS and mGPS in other cancer types and identifying higher number of patients with poor prognosis. However, the HS-GPS developed for prediction of prognosis in CRC or CC patients has not been reported in detail [5]. Furthermore, there are no reports on the value of HS-mGPS in predicting long-term prognosis in patients with colorectal cancer. Therefore, this study aimed to calculate the prognostic value of preoperative HS-GPS in patients with advanced resectable CC and compare it with those of GPS and mGPS.

## Methods

### Patients

We retrospectively examined the data of 636 consecutive patients with advanced resectable CC who underwent surgery at the Tokyo Medical University Hospital between 2000 and 2015. Of these, 41 patients with missing test data, severe obstructive enteritis or gastrointestinal perforation requiring emergency surgery, or organ failure (including patients on dialysis and those having liver cirrhosis) were excluded. In our study, though 31 patients had obstructive enteritis, we inserted a transanal tube or stent and performed surgery after a waiting period of 10 to 22 days; consequently, these cases were included in the study. The remaining 595 patients were divided into groups based on HS-GPS values. The CC stage was classified according to the eighth edition of the UICC TNM classification system. Patient blood test results analysed in this study used those from the samples that were obtained shortly before the surgery, most of which were collected 1 or 2 days before surgery. All patients underwent curative surgery.

This study was approved by the institutional review board of Tokyo Medical University Hospital.

### Postoperative follow-up

After surgery, patients were followed up every 3 or 6 months for 5 years or longer with detailed examination that included blood sampling, imaging, and endoscopy assessments. These examinations and postoperative adjuvant chemotherapy (ADJ) were performed at our institution according to the Japanese Society for Cancer of the Colon and Rectum (JSCCR) guidelines for each age [6]. Chemotherapy was administered to 10% of stage II patients and 64% of stage III patients. ADJ included the 5-fluorouracil-based regimen. If CRC recurred, most patients were treated according to the JSCCR guidelines [6]. Overall survival (OS) was calculated from the date of colectomy to the date of death or last follow-up. Recurrence-free survival (RFS) was calculated from the date of colectomy to the date of either recurrence or death or the last follow-up.

### Criteria of each GPS related score

The HS-mGPS was calculated based on the cut-off values of 0.3 mg/dL for C-reactive protein (CRP) and 3.5 g/dL for albumin levels. Patients with an elevated CRP (> 0.3 mg/dL) and hypoalbuminemia (< 3.5 mg/dL) were assigned a score of 2; those with an elevated CRP alone were assigned a score of 1; and patients without an elevated CRP (≤ 0.3 mg/dL), regardless of albumin levels, were assigned a score of 0 [5]. The GPS was scored by allocating one point each for elevated CRP (> 1.0 mg/dL) and hypoalbuminemia (< 3.5 mg/dL). tPatients with both, either, or none of these laboratory parameters were assigned scores of 2, 1, or 0, respectively. For the mGPS, patients with an elevated CRP (> 1.0 mg/dL) and hypoalbuminemia (< 3.5 mg/dL) were assigned a score of 2; those with an elevated CRP alone, a score of 1; and those with a normal CRP regardless of the albumin levels, a score of 0 [7] .

### Statistical analysis

Patient baseline characteristics in each HS-GPS group were compared using either the χ^2^ and Fisher’s exact tests. The association of GPS with OS and RFS was analysed using the Kaplan–Meier method and log-rank test. Multivariate Cox regression analysis was also performed. Statistical significance was set at *P* < 0.05. The Cox proportional hazards regression model was used to estimate the hazard ratio (HR) at 95% confidence intervals (CI). All statistical analyses were performed using the Statistical Package for Social Science software package (SPSS Inc., Tokyo, Japan). Receiver operating characteristic (ROC) curve analysis was performed using the EZR software package (EZR v1.51, Tokyo, Japan).

### Ethics statement

This study adhered to the principles of the Declaration of Helsinki and was approved by the Ethics Committee of Tokyo Medical University Hospital (T2019–0054). This study was approved by the institutional ethics board, and informed consent was obtained from all patients.

## Results

### Patient clinicopathological characteristics in association with GPS scores

The baseline characteristics of the 595 patients who underwent curative surgery for CC are presented in Tables 1, 2, and 3. The study sample included 362 men (65.7%) and 232 women (34.3%). The median age of the patients was 69.7 (range, 30–95) years. Table 1 shows patient characteristics according to the HS-GPS model. The HS-GPS score significantly correlated with age (*P* = 0.046), BMI (*P* = 0.039), tumour location (*P* = 0.008), surgical approach (*P* = 0.02), tumour size (*P* < 0.001), pT (*P* = 0.011), and pN (*P* = 0.016). Table 2 shows patient characteristics according to the GPS model. The GPS score significantly correlated with age (P = 0.046), BMI (P = 0.039), tumour location (P = 0.008), surgical approach (P = 0.02), tumour size (*P* < 0.001), pT (P = 0.011), and pN (P = 0.016). Table 3 shows patient characteristics in accordance with the mGPS model. The mGPS score significantly correlated with the surgical approach (*P* = 0.001), tumour size (P < 0.001), pT (*P* = 0.014), and pN (*P* = 0.17).

**Table 1:** The relationship between HS-GPS scores and clinicopathological characteristics

variable		HS-GPS 0		HS-GPS 1		HS-GPS 2		*P* Value
		(*N* = 437)		(*N* = 107)		(*N* = 52)		
**Age**	(years)							0.031
	< 75	313	52.7%	67	11.3%	29	4.9%	
	≥75	124	20.9%	38	6.4%	23	3.9%	
**Sex**								0.996
	female	171	28.8%	41	6.9%	20	3.4%	
	male	266	44.8%	64	10.8%	32	5.4%	
**BMI**								0.016
	≥18.5	363	64.7%	85	15.2%	42	7.5%	
	< 18.5	41	7.3%	20	3.6%	10	1.8%	
**tumor location**								0.084
	right side	178	30.0%	49	8.2%	29	4.9%	
	left side	259	43.6%	56	9.4%	233.9	4.9%	
**operation approach**								0.001
	open	249	42.0%	78	13.2%	38	6.4%	
	lap	187	31.5%	27	4.6%	14	2.4%	
**Tumor size** **T**	(cm)							< 0.001
	< 5	291	50.5%	45	7.8%	11	1.9%	
	≥5	134	23.3%	55	9.5%	40	5.9%	
								0.174
	T2/3	218	36.8%	56	9.4%	33	5.6%	
	T4	218	36.8%	17	8.3%	19	3.2%	
**N**								0.016
	N0	196	33.1%	67	11.3%	44	7.4%	
	N1/2/3	213	35.8%	50	8.4%	24	4.0%	
**CEA**	(ng/ml)							0.621
	< 5	276	51.4%	66	12.3%	29	5.4%	
	≥5	122	22.7%	27	5.0%	17	3.2%	
**CA19–9**	(U/ml)							0.204
	< 37	320	59.9%	79	14.8%	29	8.1%	
	≥37	79	14.8%	15	2.8%	12	2.2%	

*Abbreviation*: *HS-GPS* high sensivity-Glasgow Prognostic Score, *BMI* body mass index, *CEA* carcinoembryonic antigen; CA19–9 = carbohydrate antigen 19–9

*P*-values < 0.05 were considered to indicate statistical significance

**Table 2:** The relarionship between GPS score and clinicopathological characteristics

variable		GPS 0		GPS 1		GPS 2		*P* Value
		(*N* = 409)		(*N* = 117)		(*N* = 68)		
**Age**	(years)							0.046
	< 75	290	48.8%	81	13.6%	38	6.4%	
	≥75	119	20.0%	36	19.7%	30	11.4%	
**Sex**								0.075
	female	168	28.3%	35	5.9%	29	4.9%	
	male	241	40.6%	117	19.7%	68	11.4%	
**BMI**								0.039
	≥18.5	328	58.5%	108	19.3%	54	9.6%	
	< 18.5	48	8.6%	9	1.6%	14	2.5%	
**tumor location**								0.008
	right side	159	26.8%	60	10.1%	37	6.2%	
	left side	250	42.1%	57	9.6%	31	5.2%	
**opearation approach**								0.02
	open	236	39.8%	80	13.5%	49	8.3%	
	lap	172	29.0%	37	6.2%	19	3.2%	
**Tumor size**	(cm)							< 0.001
	< 5	279	48.4%	52	9.0%	16	2.8%	
	≥5	118	20.5%	60	10.4%	51	8.9%	
**T**								0.011
	T2/3	342	57.6%	99	16.7%	47	7.9%	
	T4	67	11.3%	18	3.0%	21	3.5%	
**N**								0.016
	N0	196	33.1%	67	11.3%	44	7.4%	
	N1/2/3	213	35.8%	50	8.4%	24	4.0%	
**CEA**	(ng/ml)							0.361
	< 5	263	49.0%	69	12.8%	39	7.3%	
	≥5	108	20.1%	35	6.5%	23	4.3%	
**CA19–9**	(U/ml)							0.588
	< 37	302	56.5%	83	15.5%	43	8.1%	
	≥37	74	13.9%	18	3.4%	14	2.6%	

*Abbreviation*: *GPS* Glasgow Prognostic Score, *BMI* body mass index, *CEA* carcinoembryonic antigen; *CA19–9* carbohydrate antigen 19–9

*P*-values < 0.05 were considered to indicate statistical significance

**Table 3:** The relarionship between mGPS score and clinicopathological characteristics

variable		mGPS 0		mGPS 1		mGPS 2		*P* Value
		(*N* = 493)		(N = 49)		(N = 49)		
**Age**	(years)							0.101
	< 75	345	58.1%	35	5.9%	29	4.9%	
	≥75	148	24.9%	14	2.4%	23	3.9%	
**Sex**								0.283
	female	198	33.3%	14	2.4%	20	3.4%	
	male	295	49.7%	35	5.9%	32	5.4%	
**BMI**								0.302
	≥18.5	404	87.8%	44	7.8%	42	7.5%	
	< 18.5	56	10.0%	5	0.9%	10	1.8%	
**tumor location**								0.153
	right side	159	26.8%	60	10.1%	37	6.2%	
	left	250	42.1%	57	9.6%	31	5.2%	
**opearation approach**								0.001
	open	287	48.4%	40	6.7%	38	6.4%	
	lap	205	34.6%	9	1.5%	14	2.4%	
**Tumor size**	(cm)							< 0.001
	< 5	317	55.0%	19	3.3%	11	1.9%	
	≥5	163	28.3%	26	4.5%	40	6.9%	
**T**								0.014
	T2/3	412	69.4%	41	6.9%	35	5.9%	
	T4	81	13.6%	8	1.3%	17	2.9%	
**N**								0.17
	N0	247	41.7%	27	4.6%	33	5.6%	
	N1/2/3	245	41.3%	22	3.7%	19	3.2%	
**CEA**	(ng/ml)							0.629
	< 5	312	58.1%	30	5.6%	29	5.4%	
	≥5	137	25.5%	12	2.2%	17	3.2%	
**CA19–9**	(U/ml)							0.274
	< 37	365	68.4%	34	6.4%	29	5.4%	
	≥37	87	16.3%	7	1.3%	12	2.2%	

*Abbreviation*: *mGPS* modified-Glasgow Prognostic Score, *BMI* body mass index, *CEA* carcinoembryonic antigen; *CA19–9* carbohydrate antigen 19–9

*P*-values < 0.05 were considered to indicate statistical significance

Recurrence was classified as distant recurrence (DR) and local recurrence (LR). LR was defined as any clinical or histological evidence of tumour regrowth near the primary site. DR was defined as all recurrence types except those classified as LR. Among 88 cases (20.1%) of recurrence with an HS-GPS score of 0 or 1, the initial recurrence type was DR in 20 cases (liver, 37; lung, 23; lymph node, 7; peritoneal, 9; other type, 8) and LR in 18 cases. Among 14 cases (26.9%) of recurrence with an HS-GPS score of 2, the initial recurrence type was DR in 15 cases (liver, 6; lung, 5; lymph node, 1; peritoneal, 1; other type, 2) and LR in 2 cases. There were no significant differences between any of the groups. Treatment after recurrence was performed according to JSCCR guidelines [6].

### Association of the various GPS scores and other clinicopathological factors with OS

**Fig. 1:**
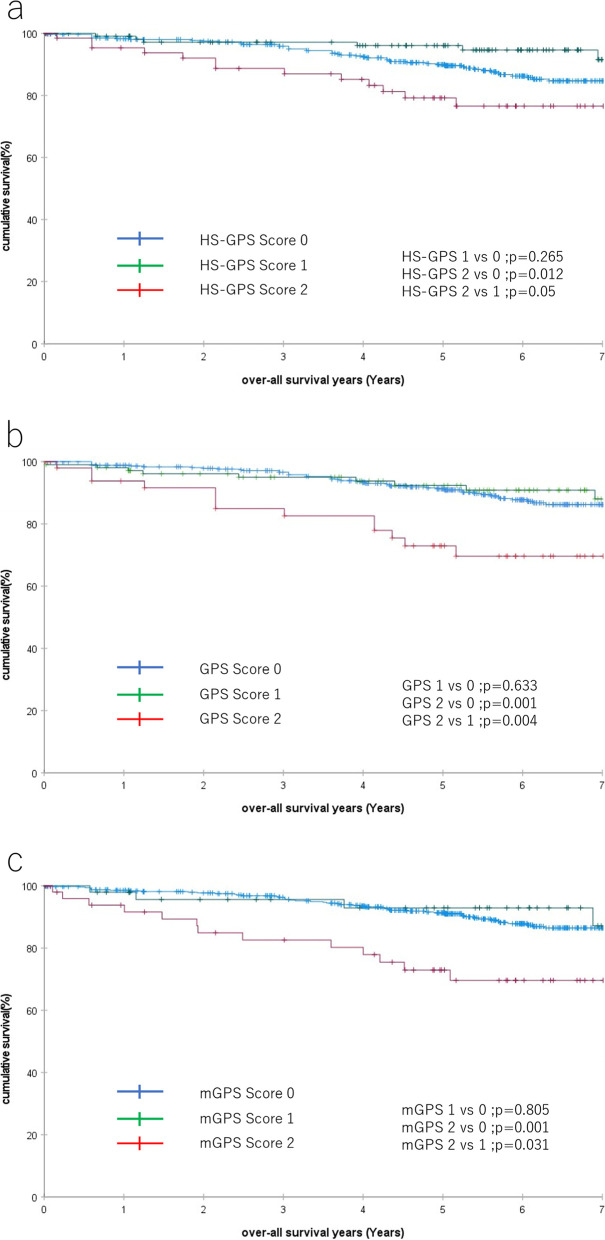
Overall survival in patients with colorectal cancer according to HS-GPS (**a**), GPS (**b**), and mGPS (**c**). GPS, Glasgow prognostic score; HS-GPS, high-sensitivity mGPS; mGPS, modified Glasgow prognostic score

Kaplan–Meier survival analysis was performed to assess the differences between the HS-GPS, GPS, and mGPS in evaluating OS. The log-rank test showed that HS-GPS 2 was a poor prognostic factor compared to HS-GPS 0 (HS-GPS 2 vs. 0; *P* = 0.012) and HS-GPS 1 (HS-GPS 2 vs 1; *P* = 0.05) (Fig. 1a). For the GPS, the test showed a significant difference in OS between GPS 2 vs 0 (*P* = 0.001) and 2 vs 1 (*P* = 0.04) (Fig. 1b). Further analysis for the mGPS showed a significant difference in OS between mGPS 2 vs 1 (P = 0.001) and 2 vs 1 (*P* = 0.031) (Fig. 1c). Of the three inflammation-based prognostic scores, a score of 2 was shown to be a poor prognostic factor.

The area under the curve (AUC) values of GPS, mGPS, and HS-mGPS for predicting OS were 0.540, 0.528, and 0.51, respectively. The AUC value of GPS was significantly higher than that of HS-GPS (*p* = 0.007). There was no significant difference in the AUC values between GPS and mGPS (*p* = 0.407), or mGPS and HS-GPS (*p* = 0.179).

Table 4 shows the results of the univariate and multivariate analyses of the predictive impacts of the three inflammation-based prognostic score models and other clinicopathological factors of OS. Univariate analysis showed that age, BMI, pT factor, pN factor, CEA, and CA19–9 were significantly different in OS prediction. Multivariate analysis was performed using the factors that showed significant differences (*P* < 0.05) in univariate analysis among inflammation-based prognostic scores and other clinicopathological factors. For the HS-GPS model, multivariate analysis confirmed that age (HR, 2.049; 95% CI, 1.195–3.413; *P* = 0.009), pT (HR, 2.254; 95% CI, 1.272–3.992; *P* = 0.005), pN (HR, 1.925; 95% CI, 1.081–3.429; *P* = 0.026), and HS-GPS 2 vs 0 (HR, 2.638; 95% CI, 1.046–6.650; *P* = 0.04) were independent prognostic predictors of OS. For the GPS model, pT (HR, 2.381; 95% CI, 1.315–4.311; *P* = 0.004), pN (HR, 2.014; 95% CI, 1.016–3.670; *P* = 0.022), GPS 2 vs 0 (HR, 1.444; 95% CI, 1.018–2.048; P = 0.04) were independent prognostic predictors. Further, for the mGPS model, age (HR, 1.98; 95% CI, 1.163–3.371; P = 0.02), pT (HR, 1.955; 95% CI, 1.090–3.508; *P* = 0.025), pN (HR, 2.04; 95% CI, 1.157–3.598; *P* = 0.014), mGPS 2 vs 0 (HR, 1.51; 95% CI, 1.066–2.140; P = 0.02) were independent prognostic predictors.

**Table 4:** Results of univariate and multivariate analysis of clinicopathological factors affecting OS

	variable	univariate analysis	multivariate analysis								
			HS-GPS model			GPS model			mGPS model		
		*p* value	HR	95%CI	*p* value	HR	95%CI	*p* value	HR	95%CI	*p* value
**Age**	(years)	0.001	2.049	1.195–3.413	0.009	2.052	1.180–3.568	0.11	1.98	1.163–3.371	0.02
	< 75										
	≥75										
**Sex**		0.764									
	female										
	male										
**BMI**		0.035	0.562	0.300–1.054	0.072	0.502	0.270–1.003	0.051	0.601	0.321–1.125	0.111
	≥18.5										
	< 18.5										
**tumor location**		0.725									
	right side										
	left side										
**operation approach**		0.895									
	open										
	lap										
**Tumor size**	(cm)	0.344									
	< 5										
	≥5										
**T**		< 0.001	2.254	1.272–3.992	0.005	2.381	1.315–4.311	0.004	1.955	1.090–3.508	0.025
	T2/3										
	T4										
**N**		0.001	1.925	1.081–3.429	0.026	2.014	1.016–3.670	0.022	2.04	1.157–3.598	0.014
	N0										
	N1/2/3										
**CEA**	(ng/ml)	0.019	1.547	0.894–2.674	0.119	1.583	0.913–2.746	1.102	1.601	0.941–2.721	0.082
	< 5										
	≥5										
**CA19–9**	(U/ml)	0.038	1.238	0.630–2.611	0.493	0.906	0.421–1.948	0.8	1.13	0.561–2.276	0.733
	< 37										
	≥37										
**HS-GPS**	0	reference									
	1	0.265									
	2	0.012	2.638	1.046–6.650	0.04						
**GPS**	0	reference									
	1	0.633									
	2	0.001				1.444	1.018–2.048	0.04			
**mGPS**	0	reference									
	1	0.805									
	2	0.001							1.51	1.066–2.140	0.02

*Abbreviation*: *HS-GPS* high sensivity modified-Glasgow Prognostic Score, *GPS* Glasgow Prognostic Score, *m-GPS* modified-GPS, *BMI* body mass index, *CEA* carcinoembryonic antigen, *CA19–9* carbohydrate antigen 19–9

*P*-values < 0.05 were considered to indicate statistical significance

### Association of the various GPS scores and other clinicopathological factors with RFS

**Fig. 2:**
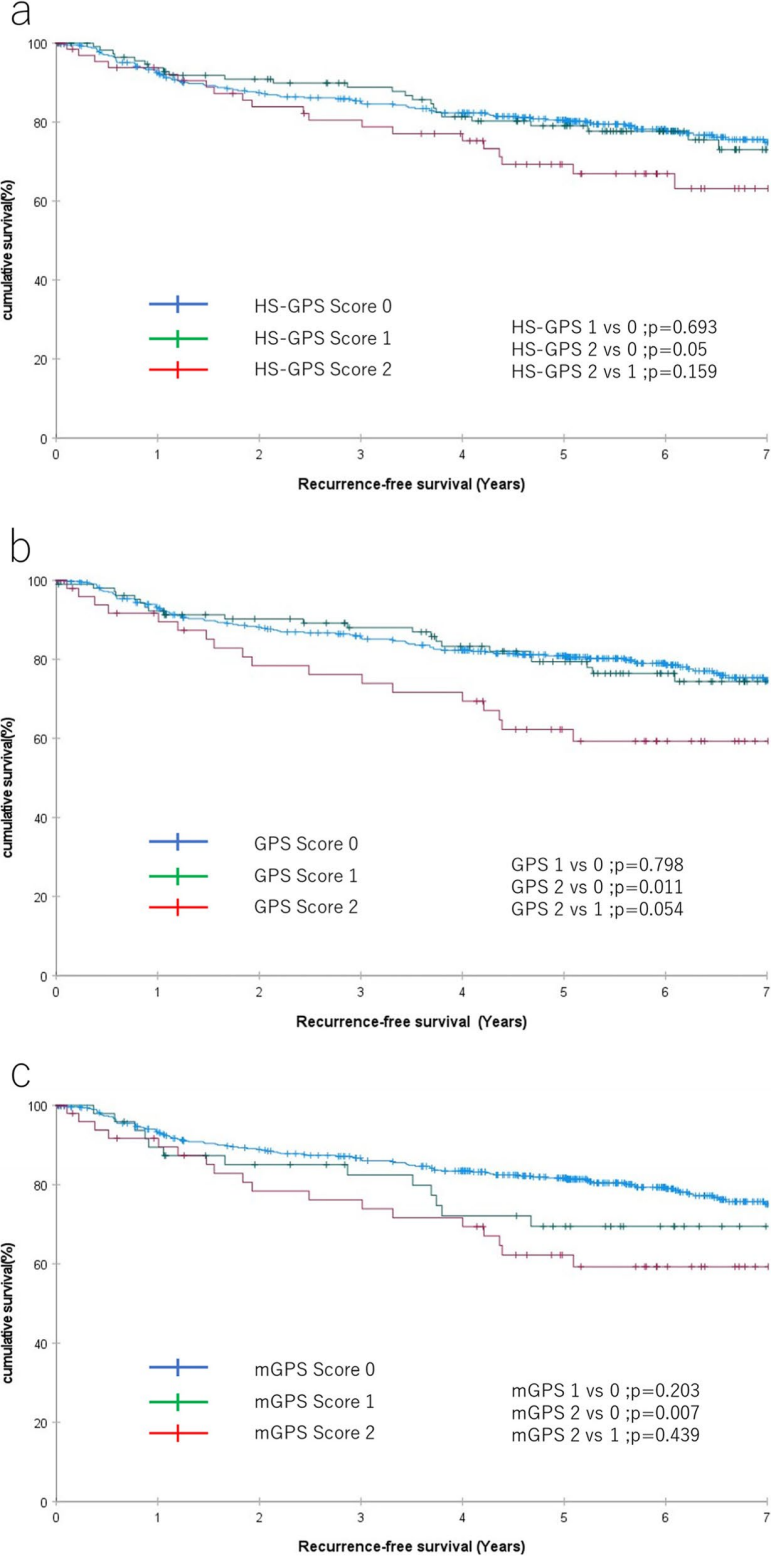
Recurrence-free survival in patients with colorectal cancer according to HS-GPS (**a**), GPS (**b**), mGPS (**c**). GPS, Glasgow prognostic score; HS-GPS, high-sensitivity mGPS; mGPS, modified Glasgow prognostic score

Kaplan–Meier survival analysis was performed to assess the differences between the HS-GPS, GPS, and mGPS in evaluating RFS. The log-rank test did not show a significant difference in RFS between HS-GPS 2 vs 0 (*P* = 0.05) (Fig. 2a). For the GPS, the test showed a significant difference in GPS 2 vs 0 (*P* = 0.011) (Fig. 2b). For the mGPS, the test showed a significant difference in mGPS 2 vs 0 (*P* = 0.007) (Fig. 2c).

Table 5 summarises the results of the univariate and multivariate analyses of the predictive ability of each inflammation-based prognostic score model and other clinicopathological factors for RFS. Univariate analysis showed that age, pT factor, pN factor, and CEA showed significant differences in RFS prediction. Multivariate analysis was performed for the factors that showed significant differences (*P* < 0.05) in the univariate analysis among inflammation-based prognostic scores and other clinicopathological factors. In the multivariate analysis of the HS-GPS model, pN (HR, 2.747; 95% CI, 1.797–4.199; *P* < 0.001) and CEA (HR, 2.093; 95% CI, 1.420–3.086; P < 0.001) were independent prognostic predictors of RFS. For the GPS model, multivariate analysis showed that pN (HR, 2.549; 95% CI, 1.648–3.857; P < 0.001), CEA (HR, 2.093; 95% CI, 1.420–3.086; P = < 0.001), and GPS 2 vs 0 (HR, 1.537; 95% CI, 1.190–1.987; *P* = 0.002) were independent prognostic predictors of RFS. In the analysis of the mGPS model, pN (HR, 2.55; 95% CI, 1.715–3.793; P < 0.001), CEA (HR, 1.998; 95% CI, 1.376–2.902; P < 0.001), and mGPS 2 vs 0 (HR, 1.555; 95% CI, 1.206–2.005; *P* = 0.004) were independent prognostic predictors.

**Table 5:** Results of univariate and multivariate analysis of clinicopathological factors affecting RFS

	variable	univariate analysis	multivariate analysis								
			HS-GPS model			GPS model			mGPS model		
		*p* value	HR	95%CI	*p* value	HR	95%CI	*p* value	HR	95%CI	*p* value
**Age**	(years)	0.027	1.349	0.897–2.028	0.151	1.281	0.848–1.934	0.24	1.232	0.830–1.830	0.3
	< 75										
	≥75										
**Sex**		0.545									
	female										
	male										
**BMI**		0.174									
	≥18.5										
	< 18.5										
**tumor location**		0.912									
	right side										
	left side										
**operation approach**		0.078									
	open										
	lap										
**Tumor size**	(cm)	0.086									
	< 5										
	≥5										
**T**		< 0.001	1.571	0.777–3.179	0.209	1.572	0.78–3.618	0.206	1.555	0.802–3.014	0.191
	T2/3										
	T4										
**N**		0.001	2.747	1.797–4.199	< 0.001	2.549	1684–3.857	< 0.001	2.55	1.715–3.793	< 0.001
	N0										
	N1/2/3										
**CEA**	(ng/ml)	< 0.001	2.093	1.420–3.086	< 0.001	2.093	1.420–3.086	< 0.001	1.998	1.376–2.902	< 0.001
	< 5										
	≥5										
**CA19–9**	(U/ml)	0.064									
	< 37										
	≥37										
**HS-GPS**	0	reference									
	1	0.693									
	2	0.051									
**GPS**	0	reference									
	1	0.798									
	2	0.011				1.537	1.190–1.987	0.002			
**mGPS**	0	reference									
	1	0.203									
	2	0.007							1555	1.206–2.005	0.004

*Abbreviation*: *HS-GPS* high sensivity modified-Glasgow Prognostic Score, *GPS* Glasgow Prognostic Score, *m-GPS* modified-GPS, *BMI* body mass index, *CEA* carcinoembryonic antigen, *CA19–9* carbohydrate antigen 19–9

*P*-values <0.05 were considered to indicate statistical significance

## Discussion

CRC prognosis is based on the UICC TNM classification; however, differences in outcomes have been reported among patients presenting with the same disease stage [8, 9]. CRC has different treatment strategies and prognoses owing to various characteristics such as stage, localisation (right or left; colon or rectum), and genotype. Therefore, biomarkers using nutritional or immunological factors associated with cancer progression and carcinogenesis have been established for improving prediction accuracy; furthermore, it is more useful to perform classification that considers each stage and localisation [10–12]. In CRC, which has a variety of treatment strategies, surgery is the mainstay of almost all approaches to resectable advanced. We considered that this group of patients was the most suitable for evaluating the biological characteristics of CRC.

Forrest et al. reported on the GPS model as a cumulative score of CRP and albumin levels, showing a significant prognostic effect in patients with lung cancer [13]. Subsequently, McMillan et al. reported that the mGPS predicted CRC patient prognosis more accurately [14]. Prediction of prognosis for CRC or other cancers using these scores has already been reported, including meta-analysis studies [2, 15, 16].

The accuracy of the three inflammation-based prognostic scoring systems is a frequent topic of discussion. GPS scores are determined using both CRP and albumin values; a score of 0 in both the mGPS and HS-mGPS systems is determined using CRP alone, regardless of albumin levels. Therefore, though hypoalbuminemia is more likely to occur secondary to elevated CRP levels, a crucial difference between the GPS and mGPS or HS-GPS is the inclusion of patients with hypoalbuminemia in the absence of elevated CRP levels. Thus, both the inflammatory response and nutritional status must be considered to predict the prognoses of cancer patients more accurately [17]. Our study was consistent with past reports. Though HS-GPS was able to predict the prognosis by evaluating the OS, it was not an independent marker of prognosis prediction for cancer recurrence by evaluating RFS. A disadvantage of the mGPS scoring system is that patients with abnormal values are a minority, and studies may only select certain minority patients with an unfavourable prognosis, which may introduce a bias. To address these problems, Proctor et al. confirmed that the HS-mGPS could enhance the prognostic values of the GPS and mGPS in a large cohort of 12,119 patients with cancer [5]. However, the number of patients with CRC in this study was limited, and the patients examined had significantly different clinicopathological classification among different cancer types. There is an increasing evidence that the inflammatory indices of HS-GPS play important roles in predicting survival in many cancers. However, Hirahara et al. reported that GPS was more reliable in evaluating prognosis in gastric cancer than HS-GPS, and the true effectiveness of HS-GPS needed to be verified in each cancer [17]. There are currently no reports analysing the prognostic impact of HS-GPS in CRC alone. Our study is the first to investigate the effectiveness of HS-GPS in patients with CRC. In our study, it was possible to predict the long-term prognosis using HS-GPS in a limited way; however, and it was not useful in predicting long-term prognosis as that using GPS. This is similar to the results of a study by Hirahara et al. on gastric cancer, and the effectiveness of HS-GPS in predicting prognosis in CRC may be limited.

This study has several limitations. First, selection bias may have been introduced because this was a single-institution, retrospective study. Though our study did not demonstrate that HS-GPS was useful in assessing long-term prognosis using RFS, we did determine that the HS-GPS score 2 group tended to have a poor prognosis. This suggests that having a larger sample size may provide useful results. Second, we did not consider ADJ. Approximately 30% of patients receive ADJ, but it is difficult to analyse the specific regimen used and other associated parameters. Chemotherapy used for CRC varies by country and age, and the choice of the multitude of regimens available serves as a prognostic factor. In particular, elderly and undernourished patients may have difficulty tolerating anticancer drugs, and it is difficult to completely eliminate this effect. Third, the relationship with other markers of systemic inflammation and nutrition is unknown. Further improvement in the prognosis of CRC may not be possible with a single marker.

## Conclusion

HS-GPS was useful for predicting the OS prognosis of resectable advanced CC. Long-term prediction of RFS only showed a dominant tendency. HS-GPS may be less effective as a prognostic score for CC than GPS.

## Data Availability

The datasets during and/or analysed during the current study are available from the corresponding author upon reasonable request.
